# Subcomplex Iλ Specifically Controls Integrated Mitochondrial Functions in *Caenorhabditis elegans*

**DOI:** 10.1371/journal.pone.0006607

**Published:** 2009-08-12

**Authors:** Marni J. Falk, Julie R. Rosenjack, Erzsebet Polyak, Wichit Suthammarak, Zhongxue Chen, Phil G. Morgan, Margaret M. Sedensky

**Affiliations:** 1 Division of Human Genetics, Department of Pediatrics, The Children's Hospital of Philadelphia and University of Pennsylvania, Philadelphia, Pennsylvania, United States of America; 2 Department of Anesthesiology, Case Western Reserve University School of Medicine, Cleveland, Ohio, United States of America; 3 Biostatistics & Data Management Core, The Children's Hospital of Philadelphia, Philadelphia, Pennsylvania, United States of America; 4 Department of Anesthesiology, Seattle Children's Regional Medical Center and University of Washington, Seattle, Washington, United States of America; Universidade de Sao Paulo, Brazil

## Abstract

Complex I dysfunction is a common, heterogeneous cause of human mitochondrial disease having poorly understood pathogenesis. The extensive conservation of complex I composition between humans and *Caenorhabditis elegans* permits analysis of individual subunit contribution to mitochondrial functions at both the whole animal and mitochondrial levels. We provide the first experimentally-verified compilation of complex I composition in *C. elegans*, demonstrating 84% conservation with human complex I. Individual subunit contribution to mitochondrial respiratory capacity, holocomplex I assembly, and animal anesthetic behavior was studied in *C. elegans* by RNA interference-generated knockdown of nuclear genes encoding 28 complex I structural subunits and 2 assembly factors. Not all complex I subunits directly impact respiratory capacity. Subcomplex Iλ subunits along the electron transfer pathway specifically control whole animal anesthetic sensitivity and complex II upregulation, proportionate to their relative impairment of complex I-dependent oxidative capacity. Translational analysis of complex I dysfunction facilitates mechanistic understanding of individual gene contribution to mitochondrial disease. We demonstrate that functional consequences of complex I deficiency vary with the particular subunit that is defective.

## Introduction

The mitochondrial respiratory chain (RC) is crucial to multiple cellular functions including energy generation by oxidative phosphorylation (OXPHOS), reactive oxygen species generation and scavenging, calcium homeostasis, and apoptosis. Mitochondrial RC dysfunction is suspected in the setting of a broad range of findings in high energy-demand tissues, especially those with neuromuscular, cardiac, or gastrointestinal manifestations [1]. Indeed, heterogeneous mitochondrial diseases are now recognized to constitute the most common group of inborn metabolic errors, with a minimal estimated 1 in 5,000 lifetime prevalence [2], [3]. Complex I (NADH:ubiquinone oxidoreducatase, EC 1.6.5.3) is the largest and most commonly implicated RC component in human mitochondrial disease.

Complex I of bovine heart mitochondria has 45 structural subunits, 38 of which are nuclear-encoded, and 7 of which are encoded by mitochondrial DNA (mtDNA) [4]. Fourteen of the subunits have bacterial homologues and represent the catalytic “core” of the enzyme, with presumed roles in redox and proton translocation [5]. Although the three-dimensional structure of its hydrophilic domain in *Thermus thermophilus* and *Escherichia coli* has recently been solved to allow for better insight of complex I structure-function relationships [6], [7], the functions of many of the 31 supernumerary (accessory) subunits remain unknown [8]. Pathogenic mutations have now been identified in 10 of the 38 nuclear DNA (nDNA) subunits of human complex I [9], including all 7 nDNA-encoded core subunits and 3 supernumerary subunits, as well as in 3 complex I assembly factors (**Table 1**). The subunits of complex I have been grouped into three major subcomplexes based on differential centrifugation fractions in bovine mitochondria, which generally correspond to structural sublocalization: Iα comprises the the matrix arm (Iλ) plus several additional membrane bound subunits, Iβ indicates most of the membrane-bound arm, and Iγ refers to subunits having undefined location identified in “breakthrough centrifugation fraction” (4, 14).

**Table 1:** Complex I composition and *C. elegans* homology.

Subunit	Gene Name				Subcomplex	*C. elegans* Protein Similarity			
#	*E. coli*	*B. taurus*	*H. sapiens*	*C. elegans*	Localization within Bovine Complex I^a^	Protein Similarity (*H. sapiens - C. elegans*)	Predicted Homology in Wormbase	Predicted Homology in KEGG	Identified in N2 worm Mitochondria by BNG/Mass Spectrometry
1 (mtDNA)	nuoH	ND1	ND1	MTCE.11	Iγ	78.4%	+		
2 (mtDNA)	nuoN	ND2	ND2	MTCE.16	Iγ	47.9%	+		
3 (mtDNA)	nuoA	ND3	ND3	MTCE.34	Iγ	92.8%	+		
4 (mtDNA)	nuoM	ND4	ND4	MTCE.25	Iβ	88.5%	+		
5 (mtDNA)	nuoK	ND4L	ND4L	MTCE.4	Iγ	40.0%	+		
6 (mtDNA)	nuoL	ND5	ND5	MTCE.35	Iβ	64.9%	+		
7 (mtDNA)	nuoJ	ND6	ND6	MTCE.3	Iα	31.0%	+		
8	nuoG	75 kDa	NDUFS1**	*Y45G12B.1*	Iα, Iλ	97.8%	+	+	+
9#	nuoD	49 kDa	NDUFS2**	*K09A9.5*	Iα, Iλ	83.4%	+	+	+
9#	"	"	"	*gas-1 (fc21)*	Iα, Iλ	83.4%	+	+	+
10	nuoC	30 kDa	NDUFS3**	*T10E9.7*	Iα, Iλ	45.4%	+	+	+
11		18 kDa	NDUFS4**	*ZK973.10*	Iα, Iλ	72.7%	+	+	
12		15 kDa	NDUFS5	*Y54E10BL.5*	Iα	57.9%	+	+	+
13		13 kDa	NDUFS6**	*F22D6.4*	Iα, Iλ	61.4%	+	+	
14	nuoB	PSST	NDUFS7**	*W10D5.2*	Iα, Iλ	95.0%	+	+	+
15	nuoI	TYKY	NDUFS8**	*T20H4.5*	Iα, Iλ	95.8%	+	+	+
16	nuoF	51 kDa	NDUFV1**	*C09H10.3*	Iα, Iλ	96.7%	+	+	+
17	nuoE	24 kDa	NDUFV2**	*F53F4.10*	Iα, Iλ	93.7%	+	+	+
18*		10 kDa	NDUFV3		Iα, Iλ				
19*		MWFE	NDUFA1**		Iα				
20&		B8	NDUFA2	*Y63D3A.7*	Iα, Iλ	93.5%	+	+	
20&		"	"	*C25A1.13*	Iα, Iλ	[53% by blast]	No	+	
21*		B9	NDUFA3		Iα				
22*		MLRQ	NDUFA4		-				
23		B13	NDUFA5	*C33A12.1*	Iα, Iλ	73.3%	+	+	+
24		B14	NDUFA6	*Y57G11C.12*	Iα	96.2%	+	+	+
25		B14.5a	NDUFA7	*F45H10.3*	Iα, Iλ	42.3%	+	+	+
26		PGIV	NDUFA8	*Y54F10AM.5*	Iα	72.7%	+	+	+
27		39 kDa	NDUFA9	*Y53G8AL.2*	Iα	64.3%	+	+	+
28		42 kDa	NDUFA10	*K04G7.4*	Iα	73.9%	+	+	+
29*		B14.7	NDUFA11		Iα, Iλ (Iγ)				
30		B17.2	NDUFA12	*Y94H6A.8*	Iα, Iλ	90.4%	+		+
31		SDAP	NDUFAB-1	*Y56A3A.19*	Iα, Iβ	97.7%	+	+	+
32*		MNLL	NDUFB1		Iβ				
33		AGGG	NDUFB2	*F44G4.2*	Iβ	53.8%	+	+	+
34		B12	NDUFB3	*C18E9.4*	Iβ	87.4%	+	+	+
35		B15	NDUFB4	*W01A8.4*	Iα, Iβ (Iγ)	62.8%	+	+	+
36		SGDH	NDUFB5	*C25H3.9*	Iβ	71.7%	+	+	+
37		B17	NDUFB6	*ZK809.3*	Iβ	53.3%	+	+	+
38		B18	NDUFB7	*D2030.4*	Iβ	99.2%	+	+	+
39		ASHI	NDUFB8	*Y51H1A.3*	Iβ	73.5%	+		+
40		B22	NDUFB9	*C16A3.5*	Iβ	39.2%	+	+	+
41		PDSW	NDUFB10	*F59C6.5*	Iβ	54.6%	+	+	+
42		ESSS	NDUFB11	*F42G8.10*	Iβ	[39.2% by blast]	No		+
43*		KFY1	NDUFC1		Iγ				
44		B14.5b	NDUFC2	*Y71H2AM.4*	Iβ (Iγ)	61.2%	+	+	
45		B16.6	GRIM19	*C34B2.8*	Iα, Iλ	84.2%	+		
Assembly Factor		NDUFAF1	NDUFAF1**	*C50B8.3*	-	57.6%	+		
Assembly Factor		B17.2L	NDUFAF2**	*Y116A8C.30*	-	[24% by blast; 73.9% to bovine]	No		
Assembly Factor		C13H20ORF7	C20orf7**	*K09E4.3*	-	93.0%	+		
Assembly Factor		ECSIT	ECSIT	*Y17GB.5*	-	90.9%	+		

82% (31/38) of mammalian, nuclear DNA-encoded, complex I subunits demonstrate extensive homology across evolution, ranging from 41.5% to 99.2% similarity between *C. elegans* and human proteins. Mitochondrial DNA-encoded complex I subunits, although highly conserved, were not included in this study.

* indicates human subunits not conserved in *C. elegans*.

** indicates subunits in which pathogenic mutations have been identified in human patients.

# *gas-1(fc21)* missense mutant allelic to *K09A9.5* RNAi knockdown.

& two RNAi clones identified *in silico* as *NDUFA2* homologues.

a subcomplexes are defined per centrifugation fractions in bovine mitochondria, which generally correspond to structural sublocalization: Iα, matrix arm (Iλ) plus several additional membrane bound subunits; Iβ, membrane-bound arm; Iλ, matrix arm only; Iγ, undefined location identified in “breakthrough centrifugation fraction” (4, 14).

We exploited the extensive evolutionary conservation of mitochondrial proteins to undertake a global analysis of subunit contribution to complex I functions. Specifically, polarographic analysis permits assessment of mitochondrial integrated respiratory capacity from electron entry into the respiratory chain through final acceptance by oxygen, in addition to coupling between oxidation and phosphorylation, as well as inner mitochondrial membrane permeability and transporter function. Whereas null alleles of RC subunits are unlikely to produce viable animals to study, large quantities of isogenic hypomorphic *Caenorhabditis elegans* nematodes can be obtained using feeding RNA interference (RNAi) [10]–[12]. This approach permits convenient phenotypic assessments of the biological consequences of specific respiratory chain genetic defects at both the whole animal and isolated mitochondria levels to assess the effects of subunit knockdown on integrated complex I respiratory capacity as well as complex assembly [13]. We report here the relative impact of RNAi-generated gene knockdown on integrated respiratory capacity and whole animal behavior for 28 nuclear-encoded structural subunits and 2 assembly factors of complex I conserved between mammals and *C. elegans*. We show that individual subunits vary significantly in the extent to which they impact these aspects of complex I function. Deficiency of only a subset of complex I subunits is likely to result in primary mitochondrial disease, as narrowly defined by impairment of integrated respiratory capacity. We extend the correlation between complex I-dependent respiratory capacity impairment and anesthetic sensitivity [13] to specify that subunits comprising subcomplex Iλ [4], [14] in the hydrophilic, matrix arm of complex I most directly influence whole worm anesthetic behavior. Complex II-dependent respiratory capacity correlates inversely with subcomplex Iλ subunit dysfunction [15], but directly with dysfunction of subunits in membrane-bound subcomplex 1β [4], [14]. This translational approach prioritizes a relevant gene subset to investigate in human patients with impaired complex I respiration, and facilitates the study of mechanisms by which individual genes contribute to human mitochondrial RC disease.

## Results and Discussion

### *C. elegans* complex I subunit homologue knockdown studied by RNA interference

All 7 mtDNA subunits, at least 31 nDNA subunits, and 4 known complex I assembly factors demonstrate extensive evolutionary conservation between humans and *C. elegans* (**Table 1** **)**. At the individual subunit level, the extent of protein similarity by length between *C. elegans* and humans ranges between 25% and 99.2% (http://ucsc.genome.edu; www.wormbase.org; www.genome.jp/kegg). This similarity extends across all structural subcomplexes and known assembly factors (**Table 2**). 26 subunits were experimentally confirmed via mass spectrometry analysis of complex I isolated by blue native gel electrophoresis from wildtype (N2) *C. elegans* mitochondria; interestingly, no mtDNA-encoded subunits were identified by this analysis [16].

**Table 2:** General overview of human-worm complex I subunit and subcomplex homology.

	*H. sapiens*	*C. elegans*	% conserved	Studied by RNAi
Total CI subunits	45	38	84%	28
mtDNA-encoded	7	7	100%	0
nDNA-encoded	38	31 predicted *in silico*	82%	28
		(26 confirmed by BNG)		
Subcomplex of nDNA subunits				
Iλ	15	14	93%	13 (+2)*
Iα alone	8	5	63%	4
Iα, Iβ	2	2	100%	2
Iβ	11	10	91%	9
Iγ**	1	0	0%	0
undefined***	1	0	0%	0
CI Assembly Factors	4	4	100%	2

Notes:Two strains were studied for homologues of two Iλ subunits, *NDUFS2* and *NDUFA2* - see **Table 1** notes for details.

** Iγ consists of “breakthrough fractions” in the purification of subcomplexes Iα and Iβ from bovine heart mitochondria, but only *KFY1*.

Extensive conservation is apparent between human and *C. elegans* complex I composition, as broken down by either genome of origin or subcomplex. ‘Percent conserved’ indicates the number of human complex I subunits in each group for which a *C. elegans* homologue can be identified, rather than the extent of similarity between specific subunits. *, two strains each were studied for homologues of two 1λ subunits, NDUFS2 and NDUFA2, per details in **Table 1** legend. **, only KFY1 is found in no other fraction except 1γ. *** MLRQ is not localized to any subcomplex (4).

Mutations in single genes encoding several *C. elegans* mitochondrial-localized proteins have been described [17]–[19]. However, traditional genetic approaches to create and characterize such mutations are labor intensive and slow, and severe alleles are likely to result in loss of viability. We used RNAi to effectively produce animals with targeted loss-of-function in each of 28 individual, nuclear-encoded, structural subunits, and 2 assembly factors for mitochondrial complex I (**Table 3**). Young adult nematodes fed a given RNAi bacterial clone (GeneService or OpenBiosystems) for at least three generations were studied to minimize maternal effects and enhance the likelihood of consistent knockdown across worm populations. Gene knockdown in *C. elegans* RNAi studies is typically assessed only by phenotypic screen without external validation, given the well-accepted efficacy of this approach [11]. As we sought to study whether all complex I subunits had similar functional consequences, however, we applied quantitative real time PCR (qPCR) to categorically confirm whether knockdown was achieved (i.e., assess if mRNA for the intended gene target was relatively increased or decreased) in RNA isolated from F2 young adults immediately prior to mitochondrial isolation from sibling young adult worm populations (see below). As RNA was not representative of single animals under study, knockdown data were not obtained for purposes of precisely correlating phenotypic findings with knockdown extent. *Regardless, mean qPCR knockdown did not qualitatively predict either degree or direction of alterations in either respiratory capacity or anesthetic sensitivity* (**Table 3** and **Figure S1**), indicating that not all subunits equally effect these endpoints. Others have found that the extent of gene knockdown alone does not reliably predict measured phenotypic effects [20]. However, we have found that in studies of complex IV subunit (*COXIV* and *COXVa*) RNAi knockdown in *C. elegans*, the amount of RNAi knockdown was well correlated with loss of complex IV enzymatic activity [16]. Therefore, the lack of such a correlation between complex I subunits indicates they differ in their biological contributions to complex I respiratory capacity and anesthetic behavior.

**Table 3:** RNAi-generated hypomorphic *C. elegans* strains for 28 nDNA-encoded complex I subunits and 2 complex I assembly factors were studied by a gene knockdown approach in strains exposed for 3 generations to RNAi.

SUBUNIT	GENE NAME				SUBCOMPLEX	RNA INTERFERENCE		RESPIRATORY CAPACITY				COMPLEX QUANTITY		ANESTHESIA
#	*E. coli*	*B. taurus*	*H. sapiens*	*C. elegans*	Localization within Bovine Complex I^a^	RNAi Clone Source	Relative transcript knockdown in F2 worms*	% CI-dependent OXPHOS in Isolated Mitochondria*	CI OXPHOS p-value*	% CII-dependent OXPHOS in Isolated Mitochondria*	CII OXPHOS p-value*	% N2 complex I on BNG	% N2 complex V on BNG	Halothane EC_50_ in Whole Worms**
8	nuoG	75 kDa	NDUFS1	*Y45G12B.1*	Iα, Iλ	GS	55%	76%	0.130	142%	0.022	76%	40%	2.9%
9^#^	nuoD	49 kDa	NDUFS2	*K09A9.5*	Iα, Iλ	GS	51%	57%	0.022	148%	0.051	-	-	1.8%
9^#^	"	"	"	*gas-1 (fc21)*	Iα, Iλ	-	-	31%	0.000	162%	0.006	42%	53%	1.1%
10	nuoC	30 kDa	NDUFS3	*T10E9.7*	Iα, Iλ	GS	27%	72%	0.088	143%	0.088	87%	114%	2.6%
11		18 kDa	NDUFS4	*ZK973.10*	Iα, Iλ	GS	54%	88%	0.230	127%	0.170	107%	185%	2.5%
12		15 kDa	NDUFS5	*Y54E10BL.5*	Iα	OB	Ind	59%	0.011	141%	0.022	88%	94%	2.3%
13		13 kDa	NDUFS6	*F22D6.4*	Iα, Iλ	GS	26%	44%	0.006	169%	0.011	-	-	1.8%
14	nuoB	PSST	NDUFS7	*W10D5.2*	Iα, Iλ	OB	Ind	74%	0.150	100%	0.920	-	-	2.5%
15	nuoI	TYKY	NDUFS8	*T20H4.5*	Iα, Iλ	GS	78%	57%	0.022	134%	0.023	-	-	2.1%
16	nuoF	51 kDa	NDUFV1	*C09H10.3*	Iα, Iλ	GS	92%	57%	0.022	133%	0.130	-	-	1.8%
17	nuoE	24 kDa	NDUFV2	*F53F4.10*	Iα, Iλ	GS	51%	76%	0.130	147%	0.022	-	-	2.8%
20^&^		B8	NDUFA2	*Y63D3A.7*	Iα, Iλ	GS	41%	127%	0.170	137%	0.039	116%	322%	3.2%
20^&^		"	"	*C25A1.13*	"	OB	Ind	84%	0.230	94%	0.920			3.0%
23		B13	NDUFA5	*C33A12.1*	Iα, Iλ	GS	57%	90%	0.230	147%	0.011	85%	144%	2.8%
24		B14	NDUFA6	*Y57G11C.12*	Iα	GS	55%	57%	0.010	121%	0.340	85%	63%	2.2%
25		B14.5a	NDUFA7	*F45H10.3*	Iα, Iλ	OB	Ind	124%	0.230	97%	0.920	106%	134%	3.1%
27		39 kDa	NDUFA9	*Y53G8AL.2*	Iα	OB	Ind	59%	0.026	119%	0.410	-	-	1.9%
28		42 kDa	NDUFA10	*K04G7.4*	Iα	GS	58%	59%	0.022	186%	0.006	-	-	3.8%
31		SDAP	NDUFAB-1	*Y56A3A.19*	Iα, Iβ	GS	50%	75%	0.088	131%	0.130	79%	98%	2.9%
33		AGGG	NDUFB2	*F44G4.2*	Iβ	OB	Ind	56%	0.011	117%	0.170	107%	273%	2.1%
34		B12	NDUFB3	*C18E9.4*	Iβ	GS	72%	90%	0.230	142%	0.022	88%	391%	3.2%
35		B15	NDUFB4	*W01A8.4*	Iα, Iβ (Iγ)	GS	46%	48%	0.022	128%	0.230	61%	58%	3.1%
36		SGDH	NDUFB5	*C25H3.9*	Iβ	GS	21%	146%	0.011	145%	0.022	110%	46%	3.1%
37		B17	NDUFB6	*ZK809.3*	Iβ	GS	27%	69%	0.022	111%	0.660	57%	59%	4.8%
38		B18	NDUFB7	*D2030.4*	Iβ	GS	70%	77%	0.088	154%	0.130	58%	279%	3.0%
39		ASHI	NDUFB8	*Y51H1A.3*	Iβ	OB	Ind	87%	0.170	136%	0.039	117%	57%	2.9%
40		B22	NDUFB9	*C16A3.5*	Iβ	GS	55%	90%	0.170	148%	0.022	78%	290%	3.0%
41		PDSW	NDUFB10	*F59C6.5*	Iβ	GS	65%	64%	0.039	112%	0.390	90%	137%	3.2%
42		ESSS	NDUFB11	*F42G8.10*	Iβ	OB	Ind	69%	0.060	125%	0.100	90%	110%	2.8%
45		B16.6	GRIM19	*C34B2.8*	Iα, Iλ	GS	23%	53%	0.006	131%	0.290	117%	31%	2.0%
Assembly Factor		NDUFAF1	NDUFAF1	*C50B8.3*	-	GS	44%	72%	0.088	73%	0.130	117%	55%	4.1%
Assembly Factor		B17.2L	NDUFAF2	*Y116A8C.30*	-	OB	Ind	93%	0.320	132%	0.100	122%	78%	3.2%

Individual subunits significantly vary in their relative impairment of complex I-dependent OXPHOS capacity, enhancement of complex II-dependent OXPHOS capacity, alteration of respiratory complex quantity, and impact on whole worm anesthetic behavior. Subunit numbering is maintained from Table 1. qPCR confirmation of target gene RNAi knockdown in whole worm populations of each preparation is presented as mean percent knockdown across all biological replicates relative to N2. OB, Open Biosystems. GS, GeneService. Ind, indeterminate.

* , comparison to N2 worms grown on HT115 *E. coli*. Malate and succinate were OXPHOS substrates for complex I- or II-dependent integrated respiratory capacity, respectively. Complex I and V content was studied by blue native gel (BNG) electrophoresis in strains where isolated mitochondria remained.

** , EC_50_ indicates percent anesthetic at which half of worm population was immobilized. Halothane EC_50_ for N2 is 3.2%. Reported p values were obtained by non-parametric statistical analysis.

### Polarographic analysis of subunit impact on mitochondrial respiratory capacity

**Figure 1:**
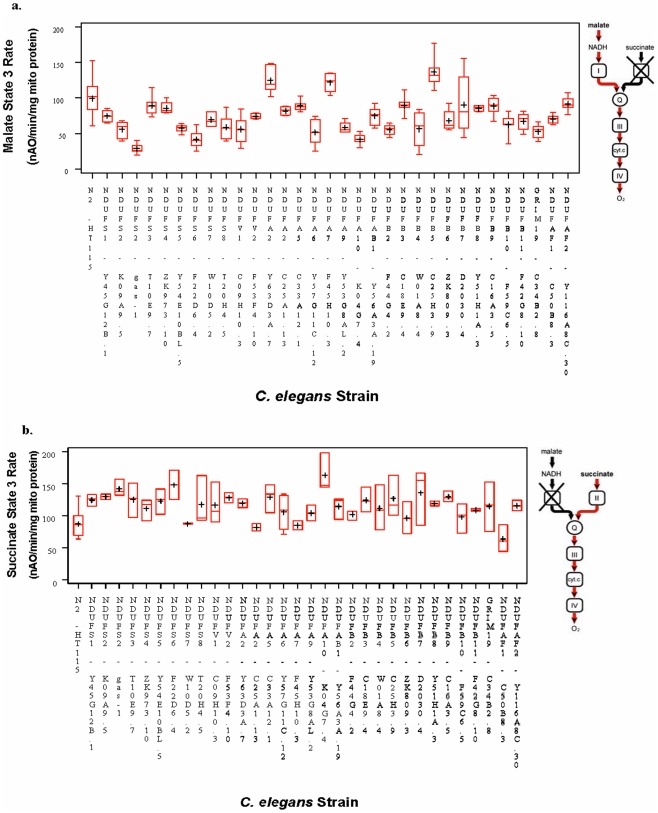
Integrated respiratory capacity of intact mitochondria from all complex I knockdown strains studied. Boxplots indicate cumulative state 3 (near-maximal) rate data from all replicates of each *C. elegans* strain interrogated with (a) a complex I-dependent substrate (malate) or (b) a complex II-dependent substrate (succinate). The length of the box represents 25^th^ to 75^th^ percentile inter-quartile range, interior cross represents mean, interior horizontal line represents median, and vertical lines issuing from the box extend to minimum and maximum values of the analysis variable. Figure insets depict specific aspects of integrated respiratory capacity interrogated with each substrate. Significance of individual strain mean differences from wildtype (N2) is detailed in Table 3.

**Figure 2:**
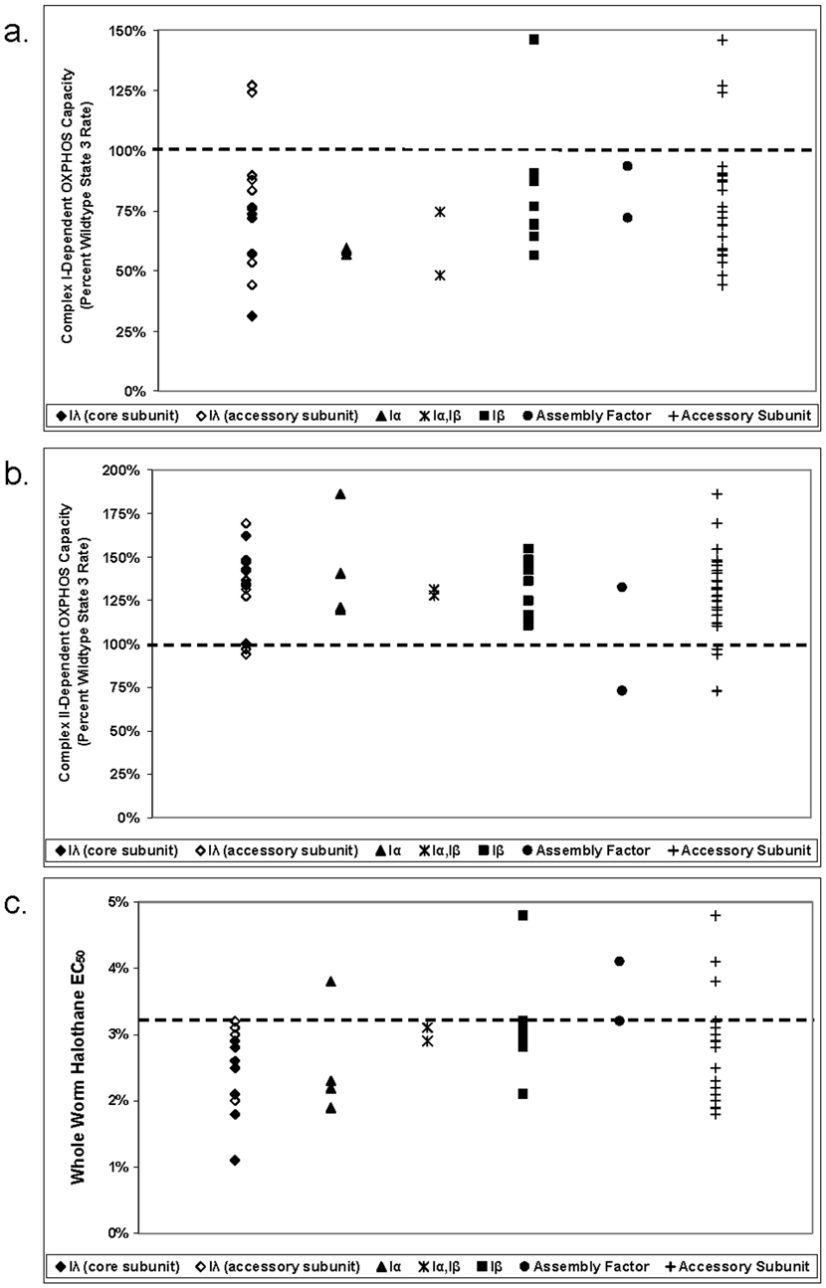
Analyses of subunit functions in *C. elegans* by known structural role. (a) Relative mean complex-I dependent respiratory capacity of each knockdown strain by subcomplex localization. (b) Relative mean complex-II dependent respiratory capacity of each knockdown strain by subcomplex localization. (c) Anesthetic behavior of each knockdown strain by subcomplex localization. Dotted lines indicate wildtype function. To discriminate subunits present in both subcomplexes 1λ and 1α from those present only in 1α, the former are labelled “1λ” and the latter “1α”. “Accessory subunits” include all structural complex I subunits studied except for 1λ core subunits.

Complex I subunit knockdown variably impact integrated respiratory function, as refers to the polarographically-assessed combined efficacy of metabolic substrate uptake and transport across the inner mitochondrial membrane, the transfer of electrons through RC complexes to oxygen, the generation of a proton gradient, and ultimately, the generation of ATP. Indeed, deficiency of only a subset of complex I subunits results in primary mitochondrial dysfunction, as characterized by significant respiratory capacity impairment (**Table 3**). Substantial variability is seen between state 3 (near-maximal) rates of biological replicates with a given substrate (**Figures 1a and 1b**), as is consistent with broad normal ranges for freshly isolated human mitochondria [21], [22]. Both the *gas-1*(*fc21*) missense allele and RNAi-generated hypomorph for the *NDUFS2* homologue (*K09A9.5*) significantly impair complex I capacity, demonstrating the validity of the knockdown technique. 15 of 28 genes when knocked down (when counting the missense and knockdown *NDUFS2* animals only once) significantly decrease complex I-dependent respiration compared to wildtype (N2) (p<0.05), with an additional 3 subunits and 1 assembly factor causing marginal impairment of complex I-dependent respiration (p<0.10) (**Table 3**). Curiously, knockdown of the *NDUFB5* homologue (*C25H3.9*) results in significantly *increased* complex I-dependent respiratory capacity. The degree of complex I dysfunction does not strictly correlate with extent of gene knockdown (**Figure S1**) or subcomplex localization (**Figure 2a**). Among the two *in silico*-predicted homologues to human subunit *NDUFA2*, neither significantly alters complex I-dependent respiratory capacity. It is possible that these two paralogues are able to substitute for each other, or that knockdown of either gene is insufficient to cause measurable changes in its function. Uncoupled rates in isolated mitochondria do not substantially differ from state 3 rates for a given strain (**Figure S2**).

**Figure 3:**
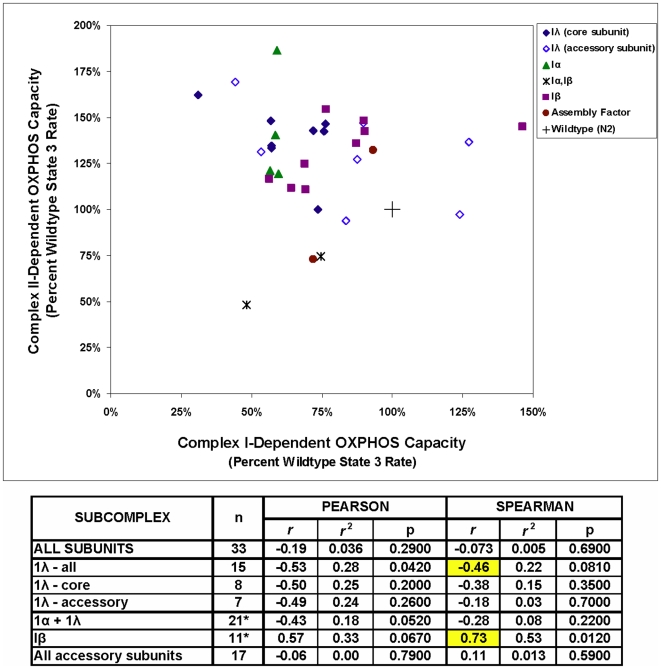
Mean complex I- and complex II-dependent respiratory capacities in *C. elegans* complex I knockdown strains. No overall significant correlation is observed between complex I dysfunction and complex II-dependent respiration. However, subcomplex analysis highlights that reduced complex I function in in membrane-bound subcomplex Iβ subunits directly correlates with reduced complex II-dependent OXPHOS capacity. In contrast, depletion of matrix arm subcomplex 1λ subunits evokes a modest compensatory increase in complex II-dependent OXPHOS capacity. See Table 1 legend for subcomplex descriptions. * The two subunits which localize both to 1α and Iβ are likely at the interface of both subcomplexes, and were therefore included in the statistical analysis for each of these subcomplexes. Results of both parametric (Pearson) and non-parametric (Spearman) analyses are shown to allow interpretation of effects of outliers and differences in subcomplex size.

Knockdown of 13 complex I subunits results in significantly (p<0.05) upregulated complex II-dependent respiration; one additional subunit increases complex II-dependent respiration but not significantly (p<0.10). (**Table 3** and **Figure 1b**), irrespective of subcomplex (**Figure 2b**). While there is no significant overall correlation between complex I- and II-dependent OXPHOS capacity (*r* = −0.19, p = 0.29), reanalysis by subcomplex localization reveals stronger and opposite correlations for subunits in the matrix versus membrane-bound subcomplexes (**Figure 3**). Dysfunction of subunits in subcomplex Iλ along the electron transfer pathway in the hydrophilic, matrix arm of complex I demonstrates a marginal, inverse correlation with complex II-dependent respiratory capacity (Pearson *r* = −0.53, p = 0.042; Spearman *r* = −0.46, p = 0.081). This presumably represents a compensatory response, as has been observed in a knockout mouse model of a subcomplex Iλ subunit, NDUFS4 (15). In contrast, dysfunction in subunits comprising the hydrophobic, membrane-bound, subcomplex 1β demonstrates a marginal, positive correlation with complex II-dependent respiratory capacity (Pearson *r* = 0.57, p = 0.067; Spearman *r* = 0.73, p = 0.012). Further subdivision into core versus accessory subunits does not significantly alter these results. Future studies are needed to elucidate the mechanism underlying this finding.

Given the predominant role of complex I in regulating respiratory control [23], we analyzed the relative impact on respiratory control of each complex I subunit knockdown strain (**Figure S3**). Mean respiratory control ratio (RCR) is significantly decreased compared to wildtype for 11 strains located across the structural subcomplexes, corresponding to human subunits *NDUFS2, NDUFS3, NDUFS6, NDUFS8, NDUFV1, NDUFA6, NDUFA10, NDUFAB1, NDUFB4, NDUFB9, NDUFB10*, and marginally decreased for *NDUFB7*. Analysis of subunit knockdown strains having relatively impaired respiratory control showed no significant correlation of RCR and mean state 3 rate (Pearson *r* = 0.30, p = 0.32).

#### RC Complex Quantitation

**Figure 4:**
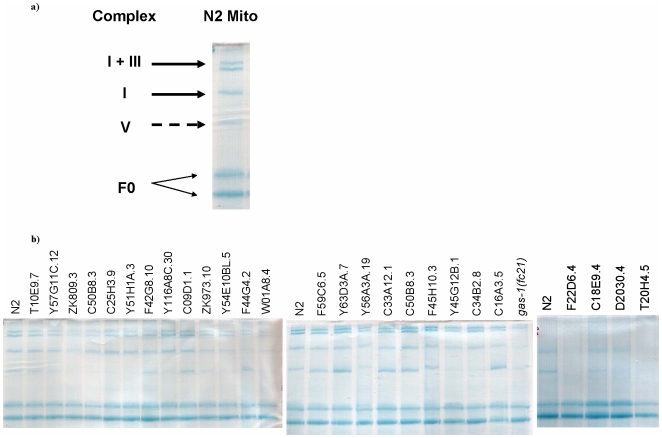
Mitochondrial blue native gel (BNG) electrophoresis in *C. elegans* complex I RNAi knockdown strains. (a) Identification of RC complexes in N2 (wildtype) mitochondria. (b) BNG electrophoresis of mitochondria isolated from RNAi-generated complex I subunit and assembly factor knockdown strains. All lanes contain 200 mg isolated mitochondrial protein, 3:1 Triton X:protein ratio, 8∶1 Dye/Triton X ratio, 4–12% gradient. Quantitation of individual complex content in each RNAi strain relative to wildtype is provided in Table 3.

Complex I subunit knockdown variably decreases the amount of complex I (**Table 3** and **Figure 4**). Observed decreases in complex I-dependent OXPHOS capacity are not predicted entirely by decreased complex I content. In other words, complex I function may be impaired without corresponding changes in the amount of assembled complex I (e.g., knockdown of the *GRIM-19* homologue (*C34B2.8*) results in 53% complex I-dependent OXPHOS capacity, but 117% complex I content when compared to the wild-type, N2). Among most subcomplex Iλ and/or Iα subunits, however, complex I content approximates polarographically-determined percent wildtype function (**Table 3**). Further analysis by organization into core versus accessory subunits permits additional insight, although interpretation is limited by sample size of core subunits in which complex I content was quantified (**Figure S4**). The two subunits localized to both bovine subcomplexes Iα and 1β (homologues of *NDUFAB1 (Y56A3A.19)* and *NDUFB4 (W01A8.4)*, plausibly localized at the interface of these two subcomplexes, directly impact both complex I-dependent (and complex II-dependent) respiratory capacity and assembly. In contrast, the only significant respiratory capacity change observed upon knockdown of two complex I assembly homologues was a marginally significant decrease in complex I-dependent OXPHOS capacity in *NDUFAF1*. However, both assembly knockdown strains have marginally increased complex I content and significantly decreased complex V content (**Table 3**). These data raise the possibility these genes are not functioning as complex I assembly factors in *C. elegans*. Indeed, complex V content appears significantly altered in many of the complex I subunit knockdown strains, although no consistent pattern is evident (**Figure 4**).

### Anesthetic sensitivity of complex I subunit knockdown strains

**Figure 5:**
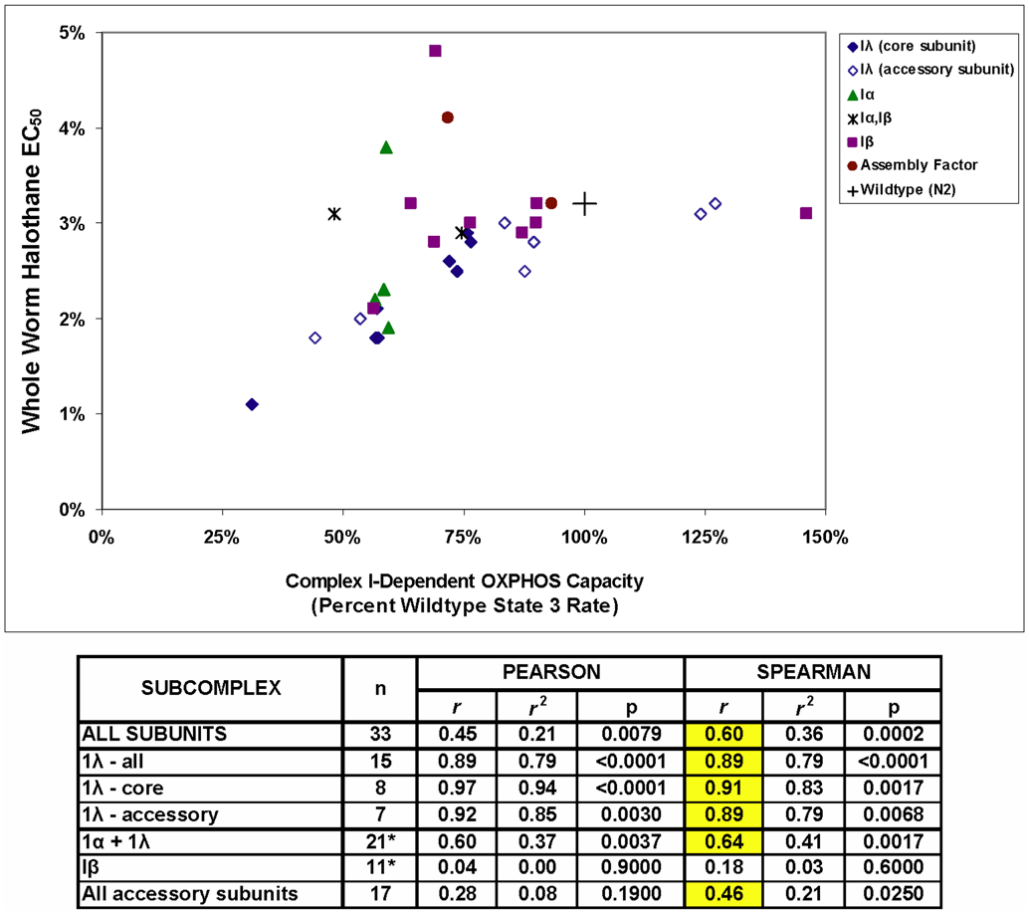
Correlation of mean complex I-dependent respiratory capacity and worm anesthetic behavior in *C. elegans* complex I knockdown strains. The overall correlation demonstrates a modest linear relationship between impaired mitochondrial complex I-dependent respiratory capacity and *C. elegans* anesthetic sensitivitiy. Subunit analysis by subcomplex localization highlights subcomplex 1λ subunits most directly influence whole animal anesthetic behavior. *As described in the legend to Figure 3, the two subunits which localize both to 1α and Iβ were included in the statistical analysis for each of these subcomplexes.

The degree of complex I-dependent OXPHOS impairment at the level of isolated mitochondria highly correlates with anesthetic sensitivity at the level of whole worms for the corresponding genetic knockdown strains (Pearson *r* = 0.45, p = 0.0079 and Spearman *r* = 0.60, p = 0.0002) (**Figure 5**). As seen in the figure, however, there are both outliers (decreased complex I rates with no decrease in EC_50_) and an apparent ceiling effect when complex I rates are increased. Thus, maximal complex I rates do not entirely predict anesthetic sensitivity, despite the overall strong correlation between these two parameters.

These data extend our previously reported correlation between complex I-dependent respiratory capacity and anesthetic sensitivity [13]. Specifically, subunit analysis by subcomplex localization reveals that the respiratory capacity of subunits in subcomplex Iλ along the electron transfer pathway in the hydrophilic, matrix arm of complex I most directly influence whole worm anesthetic behavior (Pearson *r* = 0.89, p<0.0001). The magnitude and significance of this finding remains unchanged when accounting for possible outliers and differences in number of subunits studied within each subcomplex (Spearman *r* = 0.89, p<0.0001). While subcomplex Iα includes all subcomplex Iλ subunits plus several subunits located in the membrane-bound arm (**Table 2**), the correlation falls considerably when including Iα and Iλ subunits together (Pearson *r* = 0.60, p = 0.0037; Spearman *r* = 0.64, p = 0.0017). Further breakdown of subunits into “core” versus “accessory” highlights that it is the core subunits (all part of subcomplex Iλ) that demonstrate the greatest correlation between complex I-dependent OXPHOS capacity and anesthetic behavior (Pearson *r* = 0.97, p<0.0001; Spearman *r* = 0.91, p = 0.0017). In contrast, a substantially diminished correlation (Spearman *r* = 0.46, p = 0.0250) is observed among the combined accessory subunits (located in Iα 1β, and Iλ subcomplexes), which appears driven by a clear absence of correlation between these parameters among membrane-bound subcomplex 1β subunits (Spearman *r* = 0.18, p = 0.60) (**Figure 5**).

The magnitude of anesthetic sensitivity is not predicted solely by complex I rate nor by subcomplex localization, as strains with similarly increased degrees of anesthetic sensitivity occur in multiple subunits of subcomplexes Iα and Iλ, and in a single subcomplex 1β subunit homologue (*NDUFB2*) (**Figure 2c**). In contrast, subcomplex analysis highlights three *anesthetic resistant* knockdown strains of homologues to an assembly factor (*NDUFAF1*), a subcomplex Iα subunit (*NDUFA10*), and a subcomplex 1β subunit (*NDUFB6*). Notably, no Iλ subunits cause anesthetic resistance. Future analysis of these exceptions may elucidate mechanisms underlying the role of complex I in anesthetic behavior.

This work confirms that complex I subunits variably impact both mitochondrial respiration and whole worm anesthetic sensitivity. Indeed, the extent of volatile anesthetic hypersensitivity directly correlates with the degree of complex I dysfunction in *C. elegans*, rather than with dysfunction of any other respiratory chain complex [13]. This has a direct translational correlate, as some children with mitochondrial complex I dysfunction display hypersensitivity to volatile anesthetics [24].

### Biological Significance

Significant genetic heterogeneity across dual genomes and high morbidity associated with mitochondrial RC dysfunction limits efforts to discern the pathogenic mechanisms underlying widespread phenotypic variability in human mitochondrial diseases [25], [26]. Currently available diagnostic techniques are unable to establish the genetic cause in at least 60% of suspected mitochondrial disease patients who have demonstrable biochemical abnormalities [26]. The pathogenesis of human complex I deficiency to date has involved homozygous or complex heterozygous mutations resulting in near total loss of function in 10 human complex I subunits and 3 assembly factors [27]. However, synergistic heterozygosity of dominant-negative missense mutations in two different complex I subunit genes has also been described [28]. Whereas null alleles of respiratory chain subunits are unlikely to produce viable animals to study, RNAi permits an opportunity to assess the physiologic importance of individual complex I subunits in multicellular animals [12]. Here, we exploited the extensive evolutionary conservation of mitochondrial composition and function between mammalian and *C. elegans* mitochondria to identify a biologically-relevant cadre of complex I subunits to investigate in human patients with impaired complex I respiratory capacity.

Since we were interested in physiologic consequences of complex I subunit dysfunction, we investigated integrated respiratory capacity of intact mitochondria from each complex I subunit knockdown strain by polarographic analysis of oxygen consumption, rather than spectrophotometry of *in vitro* enzyme activity. In so doing, we learned that deficiency of only a subset of complex I subunits likely results in primary mitochondrial RC dysfunction. We observed that complex II-dependent respiratory capacity is upregulated in matrix arm, subcomplex 1λ subunit dysfunction, as is consistent with a mouse knockout model of the subcomplex 1λ *NDUFS4* subunit [15]. Conversely, complex II-dependent respiratory capacity is significantly impaired upon dysfunction of membrane-bound subcomplex 1β subunits. Based upon our data, the lowest complex I-dependent maximal respiratory capacity (state 3 rate) measured in isolated mitochondria from *C. elegans* (specifically in the *gas-1*(*fc21*) missense allele of the *NDUFS2* homologue) is on the order of 30 nAO/min/milligram protein. However, given that complex II-dependent respiratory capacity is upregulated in these animals, we postulate the minimum complex I capacity necessary for survival in the setting of an otherwise normal functioning respiratory chain to be slightly lower than the measured rate in these viable nematodes. The *gas-1*(*fc21*)*;mev-1*(*kn1*) double mutant (harboring missense mutations in the *NDUFS2* complex I subunit and *SDH-C* complex II subunit homologues) is non-viable [29]. Thus, these animals cannot survive alone at this degree of complex I impairment without intact complex II respiration. While difficult to extrapolate these values to mammalian cells given the nematode ability to ferment [30], anaerobic respiration is insufficient to rescue the *gas-1*(*fc21*)*;mev-1*(*kn-1*) double mutant in complexes I and II. The minimal respiratory activity for survival in mammalian tissue is known to depend on tissue-specific thresholds [31], which in human fibroblasts correlates with a 40% reduction in complex I activity [32].

We also applied this translational genetic approach to explore the mechanism underlying the long-recognized, but poorly understood, role of complex I in whole animal anesthetic behavior. Our results strongly suggest that subunits comprising the subcomplex 1λ matrix arm involved in electron transfer through complex I most directly influence volatile anesthetic hypersensitivity. Future work will focus on discerning if this association is attributable to anesthetic exacerbation of membrane potential dissipation caused by selective impairment of complex I subunits that contribute to proton gradient flux.

In summary, the results presented here demonstrate that not all subunits of complex I contribute equally to efficient oxidative phosphorylation or anesthetic sensitivity. While impaired complex I respiration is the implicated mechanism in a large proportion of human mitochondrial diseases, the heterogeneity of clinical disease manifestations likely results from a host of secondary cellular consequences. Prior work in *C. elegans* indicates that complex I mutations exert their pathogenic effects in multiple ways, such as by impeding NADH metabolism, significantly altering expression of multiple intermediary metabolic pathways, increasing reactive species production, dissipating mitochondrial membrane potential, initiating apoptosis, and interfering with the function or assembly of other RC components [29], [33]–[37]. The functional importance of a variety of post-translational modifications of complex I subunits also remains an area of active investigation [27]. *C. elegans* presents the opportunity to discern relative contributions of complex I subunits to these plethora of mitochondrial roles in a tractable model animal.

## Materials and Methods

### Identification of complex I subunit C. elegans homologues

As complex I has no complete crystal structure elucidated in any species, the identity of the estimated 45 nDNA-encoded subunits of complex I in bovine mitochondria as determined by mass spectrometry was used as the initial reference list [4]. Each of the 45 protein sequences corresponding to the human complex I subunits was blasted in several browsers (www.homologene.com, www.wormbase.org, http://genome.ucsc.edu) against the *C. elegans* proteome to identify the closest *C. elegans* homologue (**Table 1**). Homology extent was determined in www.wormbase.org by blast based on percentage similarity along the length of the *C. elegans* protein corresponding to the longest human protein. Protein similarity of the nuclear-encoded complex I subunits and assembly factors as compiled by KEGG pathways (cel 100190) was cross-referenced against the final list (www.genome.jp/kegg). 31 complex I subunits and 2 complex I assembly factors were identified for *C. elegans* in this manner (**Table 1**). Two additional, recently-identified, human complex I assembly factors, *ECSIT* and *C20orf7*, were found to have *C. elegans* homologues by protein sequence blast (www.wormbase.org) [38], [39]. Mass spectrometry analysis performed on blue native gel electrophoresis of wildtype (N2 Bristol) *C. elegans* mitochondria (see below) independently confirmed the presence of 26 complex I subunits (**Table 1**). No *C. elegans* homologue was identifiable *in silico* for 6 complex I subunits (*NDUFA1, NDUFA3, NDUFA4, NDUFA11, NDUFB1, NDUFC1*). Although a potential *NDUFV3* homologue (*CO9D1.1*) was identified by early blast search, this was not confirmed in the most recent version of the genome browser and was therefore omitted from final analysis. *C. elegans* homologues for two human mtDNA subunits, *ND4L* and *ND6*, were not identifiable by protein sequence blast in either Wormbase or the UCSC genome browser; however, protein homology of these subunits is predicted by manual alignment to be 42% and 31% similarity by length, respectively (MacVector v10.0, Symantec, Cary, North Carolina) [40]. Subcomplex localization utilized for each subunit was that determined in complex I of bovine heart mitochondria [4], [14]. Of note, subcomplex 1α includes all subcomplex 1λ subunits comprising the matrix arm, in addition to other subunits that are present in the membrane arm (**Table 1**).

### Generation of RNAi-induced single gene C. elegans knockdown strains

N2 Bristol (wildtype) *C. elegans* were obtained from the *Caenorhabditis* Genetics center (St. Paul, Minnesota) and grown and maintained by standard culture techniques [41]. RNAi bacterial clones corresponding to 28 subunit genes and 2 complex I assembly genes identified by the *in silico* approach, as discussed above, were obtained from one of two publicly accessible libraries (GeneService, Cambridge, United Kingdom or OpenBiosystems, Huntsville, Alabama). Knockdown of the *NDUFS2* homologue, *K09A9.5*, was generated by RNAi using 1 mM isopropyl-β-D-thiogalactopyranoside (IPTG) and demonstrated similarity to the well-studied *gas-1(fc21)* missense [G(X:15,589,073)A] allele of this gene, which results in a non-conservative amino acid substitution (R290K) (18). A second *NDUFS2* homologue that is expressed in *C. elegans*, *T26A5.3*, has 95 percent identity to *K09A9.5* but is of unknown function and was not analyzed [42]. Two potential *NDUFA2* homologues (*Y63D3A.7* and *C25A1.13*) were identified, both of which were studied by RNAi. Bacterial RNAi feeding clone identity was verified by direct sequencing using a universal forward promoter specific to its library of origin. Standard RNAi feeding protocols were performed using 5 mM isopropyl-β-D-thiogalactopyranoside (IPTG) to induce transcription of double-stranded RNA [11]. Worms were grown in the presence of a given bacterial RNAi feeding clone at 20°C for two generations on agar nematode growth media (NGM) plates, transferred to liquid media for a third generation, washed clear of bacteria when most worms reached adulthood, and isolated by sucrose gradient centrifugation [13], [18]. 1–4×10^6^ worms were obtained in each of 2 to 7 (average 3) replicates per gene knockdown.

### Confirmation of RNAi-induced gene knockdown by relative qPCR

Whole worm total RNA was isolated from F2 generation (meaning 3^rd^ consecutive *C. elegans* generation fed a specific RNAi bacterial clone) *C. elegans* populations and studied by relative quantitation using SYBR green gene-specific primers for eighteen strains, as previously described [36]. For five strains where SYBR green qPCR results were ambiguous, as well as the last nine complex I subunit knockouts generated, relative quantitation was instead performed using Taqman *C. elegans* gene expression assays (Applied Biosystems), on an Applied Biosystems 7500 real time PCR system using Sequence Detection Software v.1.2.3 or v.1.2.4 (Foster City, CA). For Taqman assays, *T04C12.8* was used as the endogenous control, with consistent ΔCt of 28 to 30 in all samples analyzed. qPCR analyses on all RNA isolated from knockdown animals generated by OpenBiosystems RNAi open reading frame (ORF) clones failed due to positive RT(-) controls; cDNA from these RNAi clones initially fed to the worms could not be completely eliminated from worm total RNA, despite repeated worm washing prior to RNA isolation and aggressive DNAse treatment. Genomic DNA-based GeneService RNAi clones presented no similar problem.

### Polarographic analysis of integrated respiratory chain capacity in freshly isolated mitochondria

Freshly washed, living, adult *C. elegans* populations were immediately subjected to an isolation procedure performed on ice which involved homogenization, proteinase degradation of their outer cuticle, and differential centrifugation to collect the mitochondrial fraction, as previously described [13]. Polarographic measurement of intact mitochondrial integrated OXPHOS capacity was immediately performed utilizing a Clark-type electrode (Oxytherm, Hansatech Instruments, United Kingdom), as previously established [33]. Substrates specific to complex I (malate alone is sufficient to stimulate state 3 respiration in *C. elegans* mitochondria without supplemental glutamate, as is required in mammalian mitochondria [13]) or II (succinate) were used to obtain respiratory rate profiles: baseline, state 3 (near-maximal) in the presence of limited ADP, state 4 (ADP-depleted), high ADP (maximal) in the presence of excessive ADP, uncoupled with dinitrophenol (DNP), and cytochrome C stimulated with tetramethyl-p-phenylenediamine (TMPD) and ascorbate (**Figure S2**). Rates were calculated as nanoatomsO/minute/mg protein and expressed for comparison as percent wildtype rate (**Table 3**). Respiratory control ratios (state 3/state 4) and ADP/O ratio were calculated to assess mitochondrial coupling and efficiency, respectively.

### Anesthetic sensitivity assessment

Freshly washed F2 young adult worms from each strain were transferred to NGM plates and exposed to varying concentrations of the volatile anesthetic, halothane, to determine each strain's EC_50_ (effective concentration at which 50% of the animals are immobilized), as previously described [13]. A single observer (PGM) performed all anesthetic analyses.

### Assessment of mitochondrial complex content by blue native gel (BNG) electrophoresis, optical densitometry, and mass spectrometry

BNG [43] was performed to isolate mitochondrial respiratory complexes and supercomplexes. Capillary column liquid chromatography/tandem mass spectrometry analysis of complex I-containing bands on BNG electrophoresis of *C. elegans* N2 mitochondria was used to identify individual complex I subunits (**Table 1**). Complex content of individual BNG bands was quantified by optical densitometry in all complex I subunit knockdown strains for which isolated frozen mitochondria remained following polarographic assessment (**Figure 4b**). Specifically, 200 ug of mitochondrial protein determined by Lowry assay [44], were subject to BNG electrophoresis by modification of the technique of Wittig *et al.* [45] and Schagger *et al.* [46] using Triton X-100 with a 3∶1 detergent to protein mass ratio. Mitochondrial solubilization was performed at room temperature for 10 minutes, followed by 21,000× g centrifugation at 4°C for 20 minutes. Supernatants were collected and Coomassie blue G-250 was added to obtain an 8∶1 dye to detergent mass ratio before loading onto a 3.5–11% polyacrylamide gradient gel (Hoefer Inc, Hollister, MA). Individual bands representing complexes I, I:III, and V were quantified by optical densitometry (Multi Gauge V3.0, Fujifilm Life Science, Tokyo, Japan and Image J software, NIH) (**Figure 4a**) and normalized to the constant F0 bands of complex V (**Table 3**).

### Statistical analyses

Two-sided, non-parametric ANOVA analyses were performed in SAS version 9.1 (SAS Institute Inc, Cary, North Carolina) to compare state 3 polarographic rates for a given substrate of all RNAi-generated complex I subunit strains to N2 wildtype controls grown on HT115 *E. coli* (**Table 3**). Significance was set at p<0.10 given the small sample size of most strains each having two or three independent biological replicates. Pearson (parametric) correlation coefficients, Spearman (non-parametric) coefficients, and R-squared values were calculated in SAS version 9.1 using mean state 3 rates and mean Halothane EC_50_ from all biological replicates of a given gene knockdown (**Figures 3** **and** **5**). Parametric analyses provide greater power for small sample sizes, whereas non-parametric analysis is more robust to outliers; thus, results of both statistical analyses are provided, where relevant.

### Appendices

See Supporting Figures (S1–S4).

## Supporting Information

Figure S1 Correlation of target gene expression with complex I OXPHOS capacity for all complex I knockdown strains in C. elegans. Mean RNA knockdown of each target gene (assessed in whole worm populations) alone does not predict complex I-dependent respiratory capacity (state 3) of each corresponding mutant strain (assessed in intact mitochondria isolated from separate populations of each worm strain). Each point represents average knockdown and malate-dependent state 3 OXPHOS rate for a particular subunit from 3 replicate experiments. Apparent lack of correlation between relative transcript knockdown and complex I function may relate to limited transcriptional analyses performed due to the experimental model used. However, individual subunits appear to differ in their biologic contribution to complex I respiratory capacity. Red diamond indicates N2.
(0.01 MB PDF)

Figure S2 Compilation of isolated mitochondria complex I-dependent OXPHOS mean rates for all complex I knockdown strains in C. elegans using malate as a substrate. Uncoupled rates in the presence of dinitrophenol (DNP) are not substantially higher than respective state 3 (near-maximal ADP stimulated) or high ADP (utilizing non-rate limiting ADP concentrations) rates for each mutant. Mitochondrial viability following uncoupling is confirmed by robust TMPD plus ascorbate stimulated cytochrome C-dependent OXPHOS capacity.
(0.02 MB PDF)

Figure S3 Respiratory control analysis in complex I knockdown strains. Malate-dependent mean respiratory control ratios (RCR), defined as state 3 rate/state 4 oxygen consumption rates, in isolated mitochondria of C. elegans complex I mutants. Among the 12 complex I mutants with impaired respiratory control, no consistent or similar magnitude decrease is observed in mean state 3 rate (r = 0.56). Error bars indicate standard deviation. Asterisks indicate p<0.0015 (to account for multiple hypothesis testing), except for D2030.4 where p = 0.0016.
(0.02 MB PDF)

Figure S4 Correlation of relative mean complex I-dependent respiratory capacity and complex I content in C. elegans RNAi-generated complex I knockdown strains. An overall modest correlation is present between impaired mitochondrial complex I-dependent respiratory capacity assessed by polarography and impaired complex I content assessed by BNG electrophoresis (Pearson r = 0.50, p = 0.014). While a very strong correlation (Pearson r = 0.95, p = 0.2) is seen between complex I respiratory function and assembly among subcomplex Iλ core subunits, this does not reach significance; this may in part be based on analysis of only three subunits including the missense mutant (gas-1(fc21)), which has the greatest impairment in both respiratory capacity and content (Spearman r = 0.50, p = 0.25). Similarly, subcomplex Iλ accessory subunits do not appear to affect complex I content (Spearman r = −0.30, p = 0.62). This preliminary analysis is suggestive that core subunits may be crucial for holocomplex assembly/stability and activity, whereas accessory subunits are not. However, definitive conclusions are limited by the small number of subunits in which complex content was studied. Of note, two subunits localizing to both subcomplexes 1α and Iβ, presumably located at their interface, also appear to have a very high correlation between complex I function and content. Statistical analyses are included, as described in Figure 3.
(0.05 MB PDF)
