# Supplementary figures and images for: Subcomplex Iλ Specifically Controls Integrated Mitochondrial Functions in Caenorhabditis elegans
Source: PLoS One. 2009 Aug 12;4(8):e6607. doi: 10.1371/journal.pone.0006607 (PMC2719872; doi:10.1371/journal.pone.0006607)

Supplementary Figure 1.

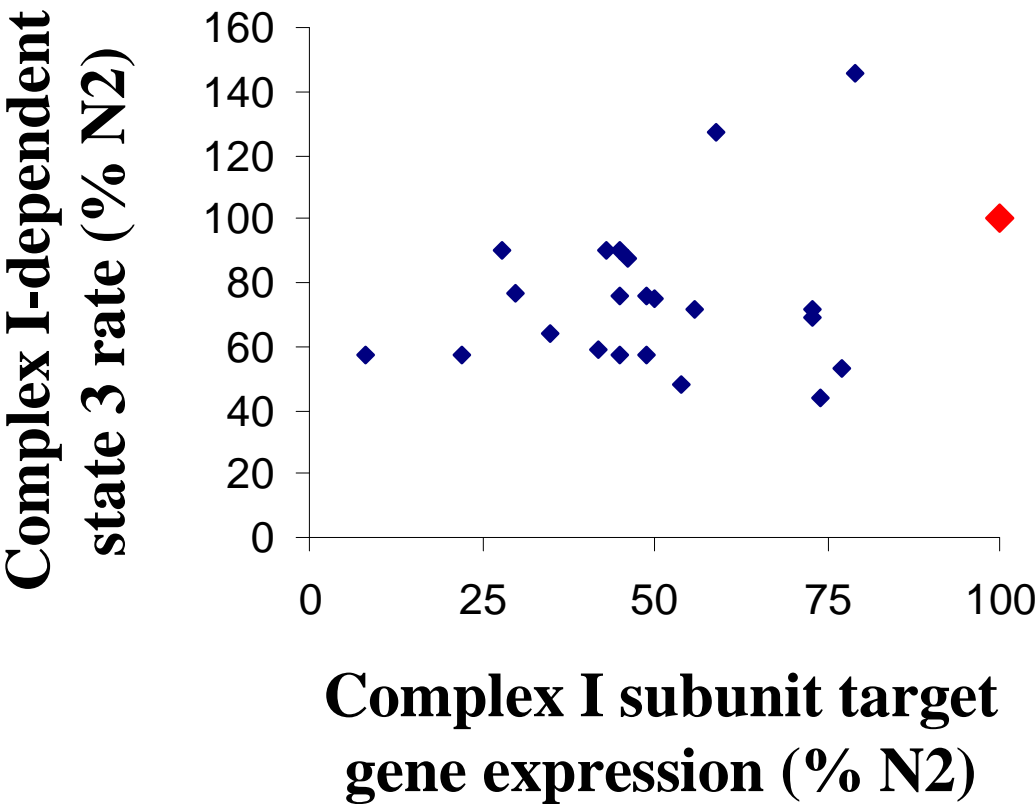

Supplement: Figure S1 — Correlation of target gene expression with complex I OXPHOS capacity for all complex I knockdown strains in C. elegans. Mean RNA knockdown of each target gene (assessed in whole worm populations) alone does not predict complex I-dependent respiratory capacity (state 3) of each corresponding mutant strain (assessed in intact mitochondria isolated from separate populations of each worm strain). Each point represents average knockdown and malate-dependent state 3 OXPHOS rate for a particular subunit from 3 replicate experiments. Apparent lack of correlation between relative transcript knockdown and complex I function may relate to limited transcriptional analyses performed due to the experimental model used. However, individual subunits appear to differ in their biologic contribution to complex I respiratory capacity. Red diamond indicates N2. (0.01 MB PDF) [file pone.0006607.s001.pdf]

Supplementary Figure 2.

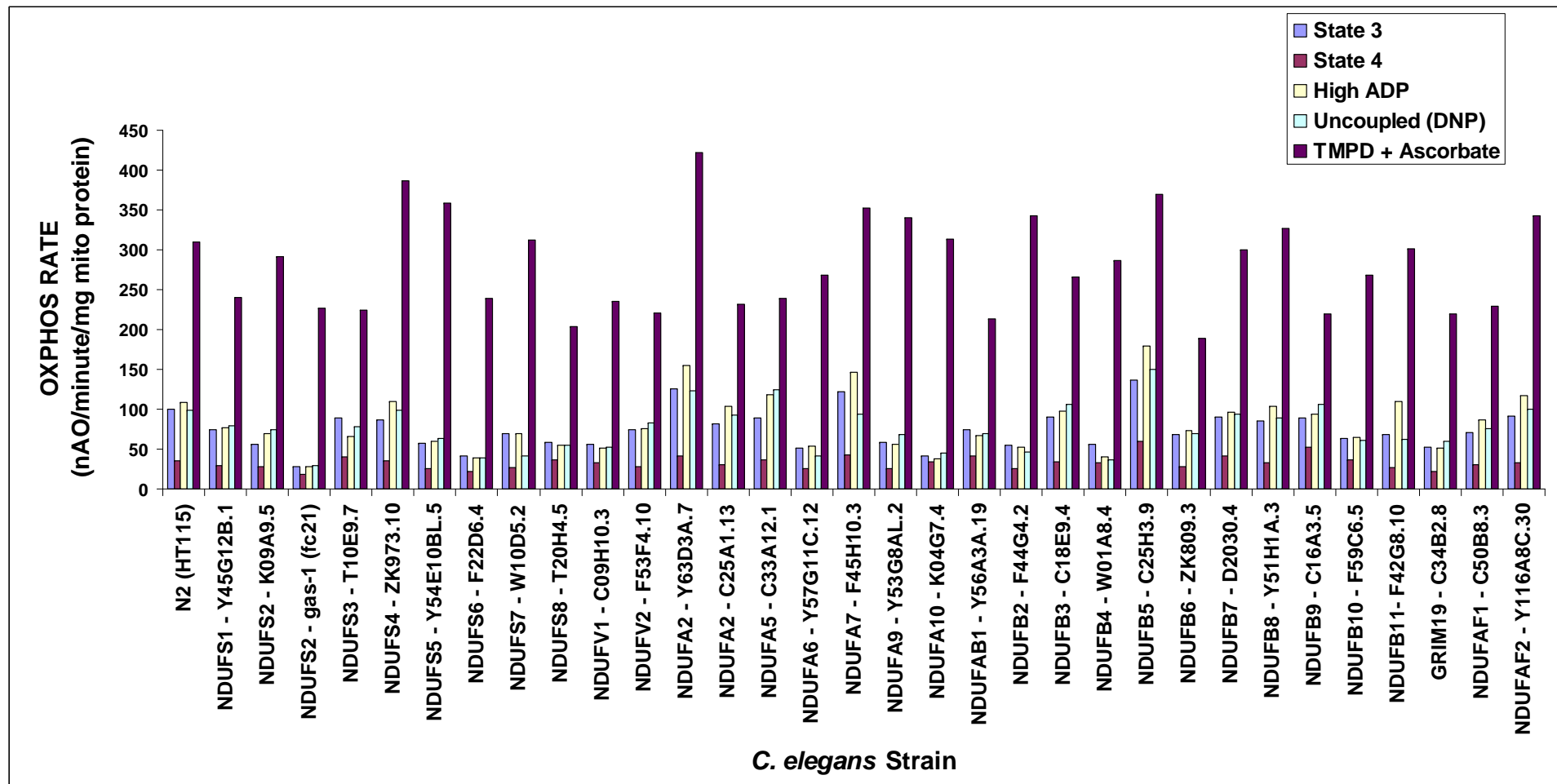

Supplement: Figure S2 — Compilation of isolated mitochondria complex I-dependent OXPHOS mean rates for all complex I knockdown strains in C. elegans using malate as a substrate. Uncoupled rates in the presence of dinitrophenol (DNP) are not substantially higher than respective state 3 (near-maximal ADP stimulated) or high ADP (utilizing non-rate limiting ADP concentrations) rates for each mutant. Mitochondrial viability following uncoupling is confirmed by robust TMPD plus ascorbate stimulated cytochrome C-dependent OXPHOS capacity. (0.02 MB PDF) [file pone.0006607.s002.pdf]

Supplementary Figure 3.

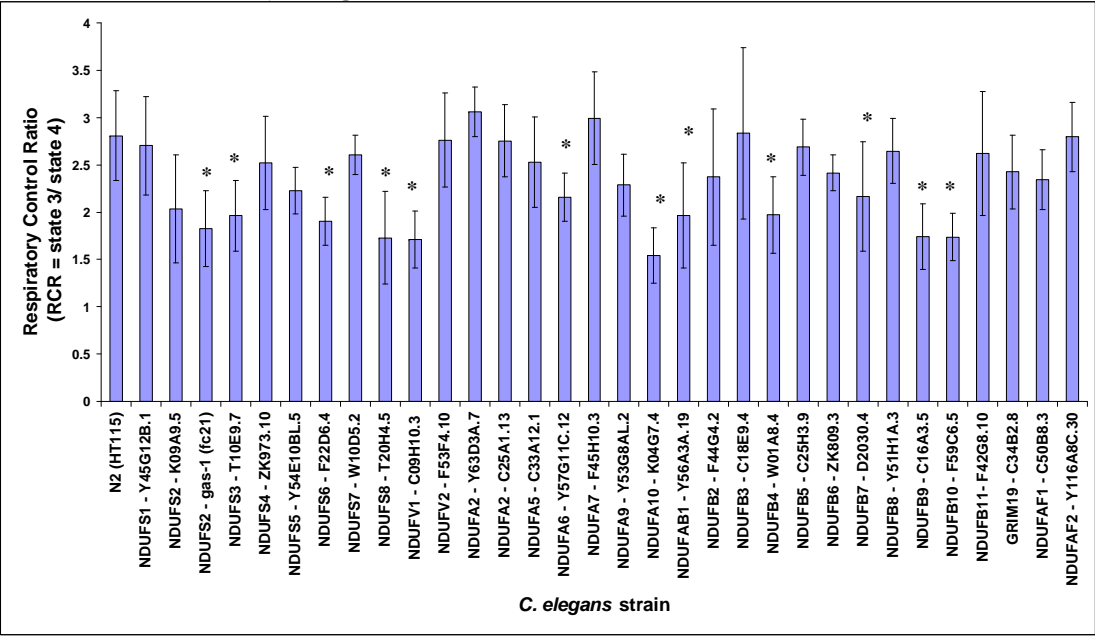

Supplement: Figure S3 — Respiratory control analysis in complex I knockdown strains. Malate-dependent mean respiratory control ratios (RCR), defined as state 3 rate/state 4 oxygen consumption rates, in isolated mitochondria of C. elegans complex I mutants. Among the 12 complex I mutants with impaired respiratory control, no consistent or similar magnitude decrease is observed in mean state 3 rate (r = 0.56). Error bars indicate standard deviation. Asterisks indicate p<0.0015 (to account for multiple hypothesis testing), except for D2030.4 where p = 0.0016. (0.02 MB PDF) [file pone.0006607.s003.pdf]
