# Supplementary material for: Subcomplex Iλ Specifically Controls Integrated Mitochondrial Functions in Caenorhabditis elegans
Source: PLoS One. 2009 Aug 12;4(8):e6607. doi: 10.1371/journal.pone.0006607 (PMC2719872; doi:10.1371/journal.pone.0006607)
Supplement: Figure S4 — Correlation of relative mean complex I-dependent respiratory capacity and complex I content in C. elegans RNAi-generated complex I knockdown strains. An overall modest correlation is present between impaired mitochondrial complex I-dependent respiratory capacity assessed by polarography and impaired complex I content assessed by BNG electrophoresis (Pearson r = 0.50, p = 0.014). While a very strong correlation (Pearson r = 0.95, p = 0.2) is seen between complex I respiratory function and assembly among subcomplex Iλ core subunits, this does not reach significance; this may in part be based on analysis of only three subunits including the missense mutant (gas-1(fc21)), which has the greatest impairment in both respiratory capacity and content (Spearman r = 0.50, p = 0.25). Similarly, subcomplex Iλ accessory subunits do not appear to affect complex I content (Spearman r = −0.30, p = 0.62). This preliminary analysis is suggestive that core subunits may be crucial for holocomplex assembly/stability and activity, whereas accessory subunits are not. However, definitive conclusions are limited by the small number of subunits in which complex content was studied. Of note, two subunits localizing to both subcomplexes 1α and Iβ, presumably located at their interface, also appear to have a very high correlation between complex I function and content. Statistical analyses are included, as described in Figure 3. (0.05 MB PDF) [file pone.0006607.s004.pdf]

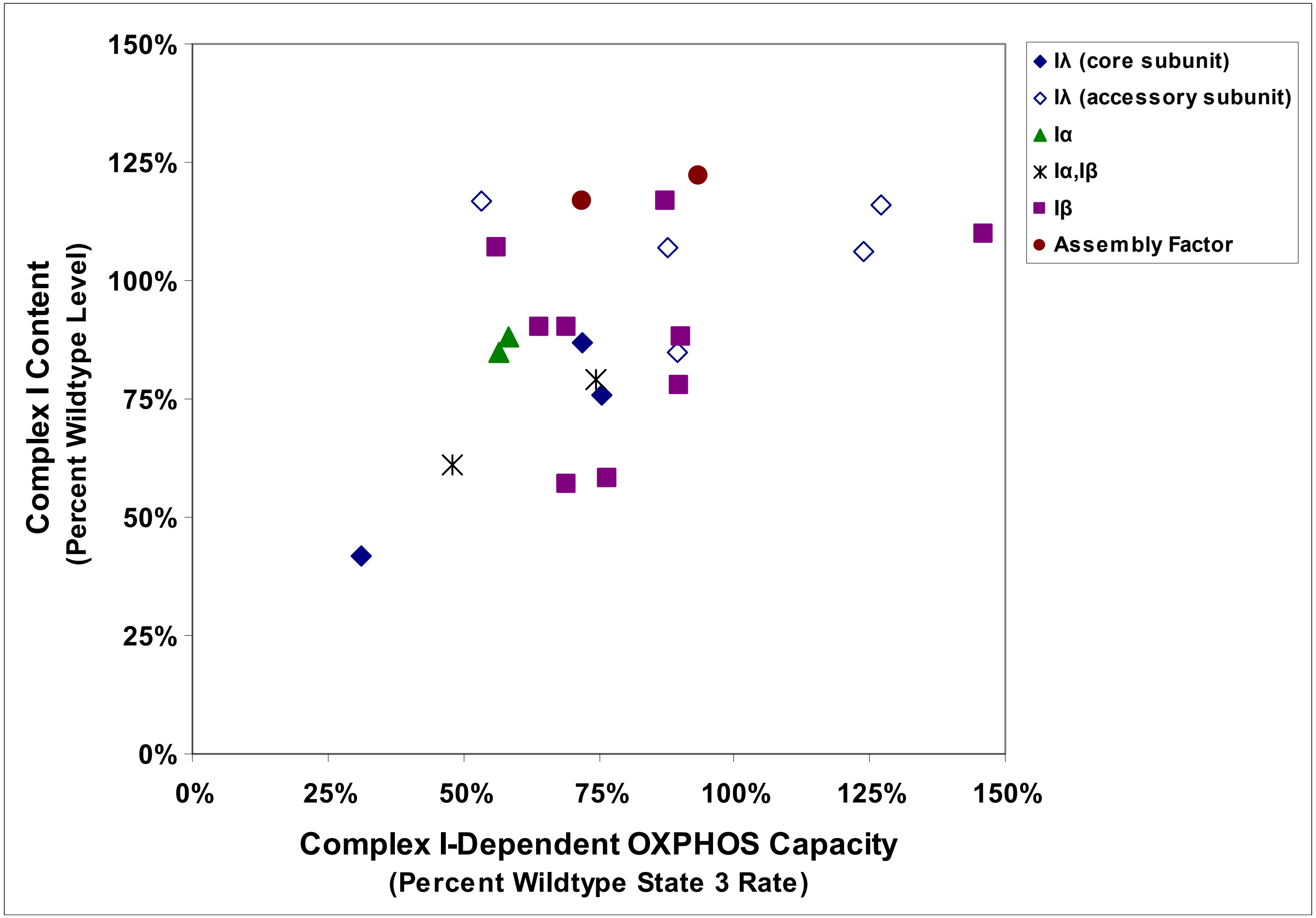

SUBCOMPLEX	n	PEARSON			SPEARMAN		
		r	r^2	p	r	r^2	p
ALL SUBUNITS (+2 A.F.)	33	0.50	0.28	0.0140	0.32	0.10	0.1300
1λ - all	9	0.64	0.41	0.0870	0.33	0.11	0.4200
1λ - core	3	0.95	0.91	0.2000	0.50	0.25	0.6700
1λ - accessory	6	-0.02	0.00	0.9700	-0.30	0.09	0.6200
1α + 1λ	13	0.67	0.45	0.0180	0.46	0.21	0.1300
Iβ	11	0.44	0.19	0.1800	0.19	0.035	0.5800
All accessory subunits	19	0.42	0.18	0.0830	0.18	0.034	0.4700
